# The Complementary and Alternative Medicine for Polycystic Ovary Syndrome: A Review of Clinical Application and Mechanism

**DOI:** 10.1155/2021/5555315

**Published:** 2021-02-26

**Authors:** Li-Yan Jia, Jia-Xing Feng, Juan-Li Li, Fang-Yuan Liu, Liang-zhen Xie, Shou-Juan Luo, Feng-Juan Han

**Affiliations:** ^1^Department of Obstetrics and Gynecology, The First Affiliated Hospital of Heilongjiang University of Chinese Medicine, Harbin, China; ^2^Heilongjiang University of Chinese Medicine, Harbin, China; ^3^Digestive Hospital of Heilongjiang Hospital, Harbin, China

## Abstract

As a reproductive endocrine disease, polycystic ovary syndrome (PCOS) has influenced billions of women during childbearing age worldwide. Owing to its complex etiology and ambiguous pathogenesis, there is still not a specific method to cure it. Clinical treatments, such as hormone therapy and surgical treatment, have side effects. Therefore, it is essential and urgent to seek alternative treatment to solve these problems. The satisfactory efficacy of complementary and alternative medicine (CAM), such as traditional Chinese medicine (TCM), immunotherapy, medicinal foods, vitamin therapy, diet therapy, psychotherapy, spa, and oxygen therapy, in treating PCOS, has aroused an increasing number of medical workers' concern and gradually become the mainstream. This paper reviews the application of CAM in the treatment of PCOS, especially from the perspective of TCM. Meanwhile, the limitations of the literature about CAM in the treatment of PCOS are mentioned and analyzed as well.

## 1. Introduction

Polycystic ovary syndrome (PCOS) is a reproductive endocrine disease characterized by menstrual disorder, infertility, and obesity, usually accompanied by insulin resistance and metabolic disorders [1]. Due to various reasons such as bad eating habits and unprecedented psychological and social pressure, its incidence has gradually increased as high as 5%–10% [2, 3]. The pathogenesis of PCOS is relatively complex, which may be closely related to genetic factors, endocrine, and specific environment [4]. It is usually diagnosed with the Rotterdam diagnostic criteria. According to the pathophysiology and clinical characteristics of PCOS, the treatment of PCOS includes exercise to lose weight, decrease androgen, and improve insulin resistance (IR), oral contraceptives to regulate the menstrual cycle, and ovulation induction. These conventional treatments, however, have problems such as adverse reactions, low compliance, poor efficacy, and even contraindications [5]. Therefore, it is urgent to find a more effective treatment for PCOS.

Complementary and alternative medicine (CAM), an alternative therapy independent of Western medicine, has been extensively used in health care systems around the world. Numerous studies have indicated that using CAM including acupuncture, Chinese herbal medicine, Tai Chi, yoga, and Qigong can also effectively treat PCOS with fewer adverse reactions. Based on this, the purpose of this review is to provide more accurate evidence for the treatment of PCOS by CAM and to analyze its underlying mechanisms.

## 2. Acupuncture

Acupuncture, an indispensable part of CAM, has been used in China for more than 3000 years. With the modernization of TCM, more and more Western countries have begun to apply it for treating and preventing diseases. Due to its superiorities, namely, ease of operation, economical and satisfactory effect, etc., acupuncture has gradually been used in the treatment of PCOS. According to literature searched at the database PubMed and CNKI, we concluded that after thousands of years of development, acupuncture has not only been limited to its original model but has gradually evolved into different forms such as acupuncture, electroacupuncture, warm acupuncture, acupoint embedding, and auricular points [6, 7]. At present, there are different mechanisms of acupuncture for PCOS summarized through considerable clinical and animal trials such as the fact that it can regulate the function of the hypothalamus-pituitary-ovarian axis (HPOA) and the metabolism, promote ovulation, and improve insulin resistance (IR) and endometrial receptivity (ER) [8, 9].

### 2.1. Acupuncture

Acupuncture refers to using the needle inserted into the patient's specific area of the body at a certain angle to cure diseases through the manipulations such as twisting, lifting, and thrusting [10]. Several studies have proven that acupuncture is beneficial in treating PCOS. A review summarized from limited evidence-based studies showed that acupuncture can be effective in improving PCOS-related symptoms, including the induction of ovulation and restoration of menstruation [11]. An animal experiment conducted by Zhang showed that acupuncture could significantly reduce the number of cystic expanded follicles and the rate of cystic expanded follicles, increase the corpus luteum, and significantly improve the ovarian morphology [12]. In addition, other studies have shown that acupuncture can reduce the incidence of PCOS by changing the distribution of specific intestinal flora, increasing the content of beneficial bacteria, and maintaining the balance between the internal and external environment of the patients [13]. Ee designed a protocol to verify their hypothesis that the mechanism of acupuncture for obese PCOS patients is improving IR [14].

Wu et al. [15] randomized 112 obese PCOS patients into the treatment group and control group. The treatment group was administrated with metformin combined with acupuncture Xiawan (CV10), Zhongwan (CV12), Guanyuan (CV4), Qihai (CV6), Tianshu (ST25), Shuidao (ST28), and bilateral Liangmen (ST21), while the control group was merely given metformin. After 3 months, they found that, in obese PCOS patients, acupuncture can effectively improve the ovarian function by regulating the luteinizing hormone (LH), the follicle-stimulating hormone (FSH), and the testosterone (T) and reducing LH/FSH, leptin (LEP), and obesity. Its mechanism may be related to the reduction of LEP [16]. Jin et al. [17] summarized 52 articles on the rules of acupuncture in PCOS patients and concluded that the most frequently used acupoints were Sanyinjiao (SP6), CV4, Zigong (EX-CA), Zhongji (CV3), and CV6. Ren [18] conducted a systematic review and meta-analysis on the treatment of PCOS with acupuncture and concluded that acupuncture can reduce HOMA-IR and BMI, promote ovulation, increase pregnancy rate, etc. while ensuring the safety of the treatment. However, due to issues such as the quality of the literature, further researches are needed to prove its effectiveness. Based on the data analysis, Yu et al. [19] outlined the rules of acupoint-selection for PCOS (Table 1)

### 2.2. Electroacupuncture (EA)

With the increasing application of acupuncture in Western countries, EA, a derivative of acupuncture, is gradually being used in the treatment of PCOS. EA refers to the combination of electric current with acupuncture to achieve the purpose of strengthening the stimulus [20]. At present, the mechanism of EA on PCOS has not been clarified. Xu G conducted an animal experiment showing that the number of immature follicles, the FSH, the LH/FSH, and the serum anti-Müllerian hormone (AMH) were decreased by EA stimulated on CV4, SP6, and ST36 [21]. Similarly, some studies have shown that EA can correct the imbalance of FSH and AMH in PCOS granulosa cells by inhibiting the excessive secretion of AMH, thus improving hyperandrogenemia and follicular development stagnation [22]. Peng et al. concluded that EA can improve IR, mitochondrial dysfunction, and ER by enhancing autophagy in PCOS-like rats [23].

Budihastuti UR randomized 44 PCOS patients into a control group and an EA group. The EA group was treated for 15 minutes per time, twice a week, for six consecutive weeks. The results show that EA can promote the growth of oocytes in PCOS patients [24]. Li [25] conducted a study on 62 PCOS patients undergoing IVF-ET and found that EA can improve the quality of oocytes. The mechanism may be as follows: EA acts on HPOA to regulate the secretion of gonadotropin-releasing hormone or increase the sensitivity of the pituitary to gonadotropin, indirectly regulate the secretion of FSH, and then improve the quality of follicles and embryos. Cui [26] also reached the same conclusion in the RCT and proposed that its mechanism may be related to the changes of the ovarian microenvironment.

### 2.3. Acupoint Catgut Embedding

Acupoint catgut embedding refers to using needles and absorbable catgut to stimulate meridians, balance yin, and yang, reconcile qi, and blood to achieve the purpose of curing diseases [27]. Acupoint catgut embedding not only has a function similar to acupuncture but can also stimulate continuous and spontaneous. The superiority of it lies in its remarkable and stable efficacy in treating disease with fewer adverse reactions. Numerous researches have confirmed the effectiveness and have elaborated the mechanism of acupoint catgut embedding in PCOS. Lin [28] conducted an animal experiment to explore the mechanism of acupoint catgut embedding in PCOS. They found that acupoint catgut embedding can significantly improve IR and increase the expression of MiR-125b in PCOS rats' ovaries. MiR-125b can reduce the high expression of ERK1 and ERK2, downregulate the abnormal activation of the MAPK/ERK pathway, restore the balance between proliferation and apoptosis of granulosa cells, and reduce the excessive synthesis of androgen by theca cells. It may be the epigenetic posttranscriptional mechanism of acupoint catgut embedding in improve IR and reproductive endocrine in PCOS. Wang [29] randomly divided 60 obese PCOS patients into acupoint catgut embedding group and acupuncture group. After treatment, hyperandrogenemia was significantly improved, and the total effective rates were 54% and 42%, respectively. There was no significant difference between the two groups, which indicates that acupoint catgut embedding has the same effect as acupuncture. Because of its time-saving and low-cost characteristics, it is worthy of further promotion in clinical practice. Yu et al. [30] analyzed the published literature and concluded that the top 10 acupoints used in acupoint catgut embedding for PCOS were ST25, CV4, ST40, SP6, GB26, BL23, CV12, ST36, CV6, and SP9. Due to the heterogeneity of the small sample size and low quality of literature, more clinical trials are needed to verify its efficacy to promote the clinical application.

### 2.4. Other Acupuncture Therapies

#### 2.4.1. Warming Acupuncture

Warming acupuncture refers to the process of keeping the needle, twisting the moxa mass around the needle handle to ignite, and transferring heat into the acupoints through the needle. Warming acupuncture is also a combination of acupuncture and moxibustion. Lin et al. [31] randomized 58 PCOS patients into a control group (using acupuncture treatment) and a treatment group (using warming acupuncture). After 3 sessions of treatment, the effective rates of the two groups were 66.67% and 44.43%, respectively. Xu's clinical trials also reached the same conclusion that warming acupuncture can treat PCOS effectively. The results showed that after treatment, the observation group was better than the control group in terms of ovarian volume, follicle number, ovulation number, pregnancy rate, etc. (*P* < 0.05) [32]. Gu [33] divided 62 PCOS patients into the obese group and nonobese group according to BMI. Both groups were treated with warming acupuncture for 3 cycles. The results show that warming acupuncture can effectively reduce T, LH, and LH/FSH, increase SOD activity, reduce MDA level, and improve oxidative stress (OS) in PCOS patients. Through comparison, we found that warming acupuncture improves the OS level of obese PCOS patients more significantly than nonobese PCOS patients.

#### 2.4.2. Auricular Points

When problems occur in certain parts of the body, it will react in the corresponding of the auricle. These parts refer to the auricular point. Auricular point is mainly used to treat various diseases by stimulating auricular point, which has been used as adjuvant therapy for a long time in China. The main methods of stimulating auricular point are acupuncture, embedding needle, bloodletting, auricular point sticking, magnetic therapy, massage, etc. [34]. Gao [35] randomized 60 patients with PCOS infertility into a treatment group (30 participants) and a control group (30 participants). The treatment group was given auricular points, and the control group was given oral Chinese herb medicine (CHM). The total effective rate of these two groups was 53.3% and 46.7%, respectively. There was no difference between the two groups. It shows that auricular points for PCOS infertility have an equal effect of CHM. Studies have also reported that the mechanism of auricular points in PCOS is that it can regulate the functions of the anterior pituitary and ovaries [36]. Compared with other CAMs, the auricular point is easy to operate and inexpensive, so it has a high acceptance rate and is worthy of promotion.

## 3. Chinese Herbal Medicine

As a part of CAM, CHM has saved thousands of Chinese people in the development of China. It started with Shen Nong, a medical scientist in ancient China. After thousands of years' exploration and practice, CHM has gradually become a major part of China's medical system. The clinical efficacy of CHM is remarkable, especially for gynecological diseases such as dysmenorrhea, infertility, and PCOS [37]. The following content mainly focuses on the clinical efficacy and mechanism of TCM monomer and TCM compound in PCOS [38, 39].

### 3.1. Chinese Herbal Monomer Extracts

#### 3.1.1. Berberine

Berberine is a quaternary ammonium salt from the protoberberine group of isoquinoline alkaloids. It can be found in plants such as *Berberis*, *Berberis aquifolium* (Oregon grape), and *Berberis vulgaris* (barberry) [40, 41]. Several studies have already reported about berberine on PCOS and summarized its mechanism including lowering blood glucose, improving IR, decreasing androgen, and affecting lipid metabolism. Meanwhile, a meta-analysis drew a conclusion that berberine showed a promising prospect in treating PCOS-IR [42].

Animal experiments showed that berberine could reduce blood glucose and improve IR by activating the PI3K/AKT pathway and inhibiting the upregulation of the MAPK pathway and glucose transporter 4 (GLUT4) [43, 44]. Zhang et al. [45] found that berberine could decrease the level of IR in PCOS-like rats by improving GLUT4, which can regulate both the PI3K/AKT and MAPK pathways. Another animal experiment conducted by Feng L et al. also confirmed that berberine could effectively decrease the blood supply and angiogenesis of ovarian tissue and improve the morphology of ovarian tissue in PCOS rats [46]. Zhang et al. [47] made a study on 20 rats to observe the mechanism of berberine on PCOS. The study confirmed that berberine could improve abnormal glucose and lipid metabolism in PCOS-like rats and regulate the level of serum steroid hormones. Berberine plays a vital role in the treatment of PCOS-related reproductive disorders. Studies about the mechanism of berberine pointed out that it could improve the gene level, upregulate the expression of ovarian CYP19a1 gene and uterine Glut4 gene, and downregulate the expression of ovarian CYP17a1 gene.

#### 3.1.2. Cryptotanshinone

Cryptotanshinone is extracted from danshen root, a Chinese herb. Studies have shown that it can significantly improve the abnormal glucose and lipid metabolism and hyperandrogenemia and can reduce the inhibitory effect of PI3K inhibitor LY294002 on the PI3K pathway in cerebral cortex nerve tissue, thereby protecting the activity of nerve cells, which may be the mechanism of cryptotanshinone in treating PCOS [48].

An animal experiment conducted by Kuang HY showed that cryptotanshinone could significantly reduce the capability of PCOS mice to modulate glucose levels in response to varying diets; meanwhile, it could increase the phosphorylation level of key molecules in the PI3K signaling pathway of ovarian granulosa cells that reduced the ovarian volume of PCOS rats. The expression levels of steroid hormone synthase STAR and CYP17 in rat granulosa cells can improve hyperandrogenism [49]. Another rat experiment showed that cryptotanshinone improved abnormal glucose and lipid metabolism in PCOS rats and reduced LH and T levels to restore the estrous cycle and ovulation of rats [50]. Studies have suggested that the effect of cryptotanshinone on PCOS may be achieved by downregulating the expression of the CYP17 gene and IR [51]. Wu et al. [52] randomized 72 PCOS patients into the observation group (36 cases) and the control group (36 cases). The observation group took Diane-35 combined with a Tanshinone capsule, while the control group was given Diane-35 merely. After 3 months, TG, TC, LDL-C, TSH, LH, ACTH, *β*-EP, Cor, and UFC were all improved. It is concluded that the Tanshinone capsule could improve the lipid metabolism of PCOS patients and regulate the function of the HPOA.

#### 3.1.3. Other Chinese Medicine Monomers

The total flavonoids of Cuscuta are extracted from the herbal medicine Cuscuta. Studies have shown that the total flavonoids of Cuscuta can reduce the ovarian index of PCOS-like rats and alleviate the proliferation; meanwhile, it can also restore the HPOA function by regulating the secretion of estrogen and androgen and inhibiting the expression of ovarian apoptotic proteins [53, 54]. Glycyrrhetinic acid, a main component of the traditional Chinese medicine licorice, can treat PCOS due to its anti-inflammatory, analgesic, antiallergic, etc. properties. Studies have indicated that the mechanism of glycyrrhetinic acid in treating PCOS lies in the fact that it can enhance insulin sensitivity and reduce testosterone in PCOS patients. In addition, glycyrrhetinic acid can increase the expression of AMPK mRNA in porcine ovarian granulosa cells and reduce the androgen secretion capacity of insulin-resistant granulosa cells and the level of CYP17 mRNA [55, 56]. Silymarin is extracted from the dried fruits of the Compositae plant *Silybum marianum*. Studies have found that both glycyrrhetinic acid and silibinin can increase the expression level of AMPK mRNA and reduce the androgen secretion capacity and CYP17 mRNA of insulin-resistant granule cells [57].

In summary, considerable trials have verified the effectiveness of Chinese medicine monomers on PCOS and concluded that its mechanism may be related to improving IR, reducing androgen level, regulating lipid metabolism, etc. However, the existing studies on PCOS by Chinese medicine monomers still lack persuasiveness due to some inevitable factors. Therefore, more high-quality research studies are needed to add weight to its efficacy in PCOS.

### 3.2. Chinese Herbal Formulas

Compared with other CAMs, formulas are the most commonly used in China. However, compared with the remarkable efficacy of formulas, the explanation of its mechanism lacks unified evidence. Therefore, the existing mechanisms of formulas in treating PCOS are still not convincing and need to be confirmed by further studies. A review about formulas in treating PCOS showed that several herbs were beneficial in improving menstrual and ovulatory dysfunctions, obesity, IR, lipid metabolism dysfunction, and androgen excess-related conditions [58]. Based on one study, the treatment of PCOS with formulas may correct the abnormal secretion of endocrine hormones and improve glucose and lipid metabolism, etc., thereby improving ovarian morphology and pregnancy outcome [59]. The usual clinical formulas mainly include Cangfu daotan Decoction, Xiaoyao San, Jiawei Xiaoyao San, and Danggui Shaoyao San. The composition of these formulas is listed in Table 2. The following content describes the treatment of PCOS individually [60].

#### 3.2.1. Cangfu Daotan Decoction (CDD)

Cangfu Daotan decoction (CDD) is used for PCOS patients, especially the patients who are the type of stagnation of phlegm and dampness. According to a rat trial conducted by Yi about CDD on obesity-type PCOS, the result showed that CDD decreased the serum levels of TCHO, TG, LDL-c, LH, T, IL-1*β*, IL-6, and TNF-*α* and increased the levels of HDL-c, FSH, and E2 in a dose-dependent manner. Meanwhile, CDD could induce the expression of OATP2B1 and OATP3A1 in ovarian and uterine tissues. Therefore, CDD could improve pregnancy outcomes [61]. Zhang and Wu [62] analyzed that the mechanism of CDD in the treatment of PCOS is to improve IR. Fan and Ling [63] randomly assigned 60 cases of obese PCOS infertility patients into two groups each of 30 cases. Both groups were given oral letrozole and progesterone, and the treatment group was given CDD on this basis. The results showed that the pregnancy rates in the treatment group and control group were 53.33% and 26.67%, respectively, with a significant difference (*P* < 0.05). In addition, it also has obvious advantages in increasing the diameter of HCG follicles and the thickness of the endometrium.

#### 3.2.2. Xiao Yao San (XYS)

Xiao Yao San (XYS) is a traditional Chinese formula that is widely used for treating gynecological disease, especially for patients with liver constraint type PCOS. The results of animal experiments by Sun et al. showed that the mechanism of XYS treatment of PCOS may ameliorate chronic unpredictable mild stress-induced irregular estrous cycles and follicles development abnormalities, a decrease of estradiol and progesterone level as well as the increase of luteinizing hormone in serum, reduce cystic follicles formation and the apoptosis and autophagy of granulosa cells, and attenuate the increase in dopamine beta-hydroxylase and c-fos level in locus coeruleus, the noradrenaline level in serum and ovarian tissue, and the expression of beta 2 adrenergic receptor in ovarian tissue. Besides, XYS alleviated the reduction of phosphorylation of ribosomal protein S6 kinase polypeptide I and protein kinase B, as well as the increase of microtubule-associated protein light chain 3-I to microtubule-associated protein light chain 3-II conversion both in vivo and in vitro [64].

#### 3.2.3. Jiawei Xiao Yao San (JWXYS)

Jiawei Xiaoyao San (JWXYS), also called Danzhi Xiaoyao San, is composed of 10 herbs, which is the most common herbal formula used for PCOS [65]. Wang [66] made an RCT to observe the effectiveness of JWXYS. 128 patients with PCOS were randomly assigned into two groups with 64 cases in each group. The control group received Ethinylestradiol cyproterone, and the treatment group was combined with JWXYS based on it. The results showed that, compared with the control group, the E2, LH, and endometrial thickness can be improved significantly in the treatment group. An animal experiment reached the same conclusion either.

#### 3.2.4. Danggui Shaoyao San (DSS)

Danggui Shaoyao San (DSS), created by Zhang Zhongjing, has been a common Chinese herbal formula, which was used since the Han dynasty. Jia and Nie [67] randomized 90 PCOS patients into the treatment group (47 cases) and the control group (43 cases). DSS was prescribed to treatment group patients, while the control group was given gonadotropin. After 6 months, the total effective rate was 85.1% and 55.8%, respectively. The results of an RCT made by Chen and Xue [68] showed that DSS could improve the sex hormones (LH, FSH) and adjust the menstrual cycle.

#### 3.2.5. Other Chinese Herbal Formulas

In addition to the commonly used Chinese herbal formula for treating PCOS mentioned above, some other formulas have been reported to be effective in PCOS. A rat study demonstrated that the underlying mechanism of Guizhi Fuling Wan in improving IR in PCOS-like rats is to regulate intestinal flora to control inflammation [69]. Heqi San, a traditional Chinese herbal formula, has been reported to regulate hormone levels in PCOS patients with metabolic disease, which may be an alternative application for treating PCOS. Zhao et al. [70] concluded through animal experiments that the beneficial effects of Heqi San on PCOS include altering serum hormone levels, recovering ovary morphological lesions, and improving IR, which are mediated through the PI3K/AKT pathway. Besides, Bushen Culuan Decoction (CCBCD) could effectively treat infertility patients with PCOS, according to a protocol of systematic review [71].

## 4. Dietary Supplements

### 4.1. Vitamin A

Vitamin A is a fat-soluble vitamin, also called retinol. Vitamin A-derived metabolites, such as retinoic acid and retinol, contribute to antioxidant activity and steroid metabolism, promote nuclear maturation of oocytes, and inhibit cell apoptosis [72–74]. A study [75] has shown that genes related to retinoic acid synthesis are expressed differently in intermembranous cells isolated from PCOS patients. A study made by Wickenheisser JK [76] showed that the use of retinol derivatives in the endometrial cells of PCOM and healthy women found that all intermembranous cells treated with trans-retinol increased dehydroepiandrosterone expression. Meanwhile, obesity and abnormal glucose metabolism are associated with retinol-binding protein 4 (rbp4) in overweight PCOS patients [77]. Another study reported that rbp4 was expressed in PCOS subcutaneous and omental adipocytes, and the rbp4 gene was upregulated, leading to changes in gonads and adrenal steroids [78].

### 4.2. Vitamin B

At present, many studies have found that vitamin B6, vitamin B12, and folic acid, which are related to homocysteine, are expressed at higher levels in PCOS patients. The reason is that homocysteine is a sulfhydryl amino acid, mainly derived from dietary methionine. Studies have pointed out that elevated HCY will increase the risk of long-term complications such as cardiovascular and reproductive symptoms in patients with PCOS [79, 80]. Folic acid, vitamin B6, and vitamin B12 play an important role in regulating homocysteine. It has been reported that in the pathophysiological study of PCOS, IR and HCY are positively correlated [81, 82]. Kaya et al. [83] confirmed that the increase in IR, obesity, and HCY levels in PCOS patients is related to the decrease of vitamin B12. Regular exercises can reduce the concentration of HCY [84]. Metformin, a sensitizer for IR, is a commonly used medicine for the treatment of PCOS. Metformin has a good effect on regulating IR, and it will reduce the levels of vitamin B12 and folic acid in the body during the treatment [85]. The elevated homocysteine levels increase the risk of cardiovascular disease in patients with PCOS.

Inositol, one of the B vitamins, is a water-soluble vitamin. Like choline, it is a lipophilic vitamin. An experiment conducted by Minozzi et al. suggested that combined contraceptives and inositol may be more effective than OCP alone in controlling the endocrine, metabolism, and clinical manifestations of patients with PCOS and may reduce insulin levels and insulin resistance [86]. Another study carried out by Genazzani et al. demonstrated that after 2 weeks of MYO administration in patients with PCOS, plasma LH, PRL, T, insulin, and LH/FSH levels were significantly reduced. All subjects with amenorrhea and oligomenorrhea resumed their menstrual cycle [87].

### 4.3. Vitamin D

Vitamin D, apart from the most well-known nonskeletal functions, has a potential role in glucose homeostasis which is connected with the secretion of insulin by pancreatic beta cells, IR in different tissues, and its influence on systemic inflammation [88]. Krul-Poel et al. [89] have confirmed that vitamin D is a significant and independent factor predicting IR, and vitamin D status is closely related to the metabolic disorder of PCOS. Other research studies showed that women with PCOS often lack vitamin D, and its concentration was lower in patients with abdominal obesity. In overweight/obese people with PCOS, vitamin D is related to fasting blood glucose and HOMA [90]. Vitamin D supplementation can reduce abnormally elevated serum AMH levels, and the reduction of serum AMH levels may improve follicular formation by reducing androgens in the ovaries of women with PCOS and increasing the sensitivity of follicles to FSH [91]. However, there are some controversial reports. After vitamin D treatment for 11 patients with PCOS for 3 weeks, there has a good effect on IR, but there is no significant change in the levels of dehydroepiandrosterone, total testosterone, free testosterone, and androstenedione [92]. Meanwhile, a recent study showed that [93] simultaneous vitamin D supplementation and low-calorie diet did not change androgen levels in overweight and obese women with PCOS, but the menstrual frequency was significantly improved.

### 4.4. Vitamin E

Vitamin E as a lipid-soluble substance with nonenzymatic antioxidant properties, also known as tocopherol, which can effectively reverse the adverse influence by oxidative stress brought to the reproductive system and endocrine system, is widely used in the field of reproductive medicine [94, 95]. Vitamin E plays a vital role in the entire reproductive process. It can antagonize the oxidative stress caused by oxygen free radicals and antioxidation imbalance and regulate the normal physiological functions of the reproductive system [96]. The combined treatment of magnesium and vitamin E for PCOS for 12 weeks has benefits for hirsutism, serum hs-CRP, plasma NO, and TAC levels [97]. Extensive research papers have proven that the treatment of vitamin E in patients with PCOS has a significant effect [98, 99]. It can significantly reduce TG and serum total cholesterol and improve IR, T, and free testosterone index in patients with PCOS. In addition, new evidence confirms that vitamin E can improve the endometrial thickness of patients with unexplained infertility but does not support the hypothesis that vitamin E can increase the ovulation rate and pregnancy rate in PCOS [100].

## 5. Tai Chi, Yoga, and Qigong

Tai Chi, a unique Chinese exercise, combines physical activity and breathing organically, allowing people to enjoy a pleasant mood while exercising. The key point of Tai Chi is concentration and slow movements. Therefore, it is suitable for the elderly with a weak constitution and chronic diseases [101]. In 2012, NEJM published an article on the treatment of Parkinson's with Tai Chi. The results confirmed that Tai Chi can alleviate the balance disorder of patients with mild to moderate Parkinson's disease, improve functional ability, and reduce falls [102]. Tai Chi can maintain the harmony of qi and blood and regulate the balance of yin and yang, which can significantly reduce BMI; risk factors reduce cardiovascular disease and improve psychological health [103–105]. Studies have shown that Tai Chi is effective for long-term complications such as obesity, cardiovascular disease, diabetes, and psychological diseases caused by PCOS [106, 107]. Meng [108] summarized that Tai Chi can promote the metabolism of cells and tissues, increase the body's utilization of glucose, the responsiveness of target cells, and the body's tolerance to glucose, prevent the composition of HbA1c, and accelerate the combination of hemoglobin and oxygen to further control blood glucose, thereby reducing the levels of FBG, HbA1c, and 2hPBG. Paul-Labrador et al. [109] believed that Tai Chi can inhibit the activation of sympathetic nerves to improve the neutral-mediated vasoconstriction of blood glucose control and reduce the glucose transport and uptake of skeletal muscle, while Tai Chi can reduce IR [110].

Yoga, a form of holistic mind-body medicine developed thousands of years ago, is a low-impact exercise that can help people maintain balance among physical, psychological, mental, sentimental, and spiritual aspects of life [111]. As a branch of CAM, it is used to treat many different diseases, such as hypertension, asthma, low back pain, arthritis and pain, stress management, and PCOS [112]. A study has shown that yoga can help control endocrine function and relieve symptoms of PCOS [113]. Compared with aerobic exercise, yoga will not cause any harm to the female reproductive system, at the same time, with the low-cost and feasible operation, so it is very promising to reduce the risk of PCOS through yoga [114]. An RCT conducted by Nidhi et al. found that yoga was more effective than conventional physical exercises in improving glucose, lipid, and insulin values, including IR values, in adolescent girls with PCOS [115]. Meanwhile, Patel et al. confirmed through RCT that regular yoga practice could reduce serum androgen in PCOS patients, which is a useful complementary therapy [112]. In addition, Ram Nidhi et al. found that a 12-week holistic yoga program was significantly better than a physical exercise program in reducing anxiety symptoms for adolescents with PCOS [116]. Ratnakumari et al. also reached a similar conclusion that yoga is efficient in bringing about beneficial changes in polycystic ovarian morphology and speculated that a longer intervention might require to regulate menstruation [117].

Although Qigong existed for thousands of years, it has never been widely adopted. In the early 1950s, Qigong became popular after Liu Guizhen advocated it. Medical Qigong, a specific form of Qigong, may have the effects of decreasing blood glucose, triglycerides, total cholesterol, body weight, BMI, and IR in type 2 diabetes in empirical and randomized controlled pilot studies [118]. An RCT conducted by Liu X has proved that after intervention by Qigong, the weight, waist circumference, leg strength, and IR improved which indicated weight reduction in the control of diabetes. Besides, a meta-analysis by Meng suggested that Qigong can improve the blood glucose of type 2 diabetes patients and benefit the management of it [119]. Owing to the fact that PCOS has the risk of obesity and cardiovascular disease, etc., we speculate that Qigong also has a certain effect in the treatment of PCOS [120].

## 6. Meditation Stress Reduction

Patients with PCOS are troubled by obesity, hirsutism, acne, irregular menstruation, and infertility, which affects their psychological health, physical condition, and quality of life to varying degrees [121]. The risk of anxiety and depression was significantly increased in patients with PCOS. Relevant research showed that more than 60% of PCOS patients were diagnosed with at least one kind of mental disorder [122]. The severity of depression, anxiety, obsessive-compulsive disorder, and somatization disorder measured by different scales in PCOS patients was higher than that in women without PCOS [123].

Emotional depression activates the neuroendocrine stress response system and simultaneously alters the immune function, leading to changes in brain structure [124, 125] and activity [126, 127]. Many people use meditation to relieve stress and treat stress-related diseases. Meditative stress reduction (MBSR) can help patients reduce emotional symptoms (e.g., anxiety, depression, and stress) and improve physical symptoms (e.g., pain) to a certain extent [128]. In addition, MBSR can reduce blood pressure, blood glucose, and inflammation [129–131]. These changes were due to improved autonomic nervous system and HPOA function after MBSR treatment [127, 132, 133]. Through MBSR treatment, PCOS patients' risk of diabetes and cardiovascular disease will eventually be reduced. Takahashi et al. [134] conducted a study on quantitatively analyzed changes in psychophysiological parameters during Zen meditation. Their findings can be an important clue to the efficient use of meditation as a therapeutic procedure. Since most of the current trials on meditation in the treatment of PCOS are small-scale, nonrandomized studies, more large-scale trials are needed to prove its efficacy on PCOS [135].

## 7. Conclusions

PCOS is a reproductive endocrine disease closely related to infertility, obesity, diabetes, cardiovascular disease, and other metabolic diseases, accompanied by different degrees of psychological disorders. CAM for PCOS mainly includes acupuncture, Chinese medicine, diet and nutrition, Tai Chi, yoga, Qigong, and meditation. Through these therapies, the incidence of cardiovascular disease and diabetes can be reduced, and anxiety and depression can be relieved as well, therefore improving the quality of life. Although CAM has been used in some countries and regions, the globalization of CAM has been hindered due to the lack of more clear research on its safety and mechanism. The specific obstacles include the following: (1) there is a lack of a larger sample size and more structured methods to evaluate the safety and pharmacological mechanism of TCM; (2) there is not enough convincing evidence to support the efficacy or safety of dietary supplements; (3) there is a lack of verification of Taijiquan, yoga, meditation, and other interventions; (4) the molecular mechanism of some CAM therapies remains to be further studied and confirmed [136]. In the future, we need larger samples and RCTs to confirm the efficacy and safety of CAM in the treatment of PCOS to provide a new method for PCOS.

## Figures and Tables

**Table 1:** The rules of acupoint-selection of acupuncture for PCOS.

Meridian	Acupoints
Ren meridian	Guan yuan (CV4), zhong ji (CV3), qi hai (CV6), zhong wan (CV12), shui fen (CV9), qu gu (CV2), dan zhong (CV17)
Spleen meridian of foot-Taiyin	San yin jiao (SP6), xue hai (SP10), yin ling quan (SP9), da heng (SP15), di ji (SP8)
Stomach meridian of foot-Yangming	zu san li (ST36), tian shu (ST25), gui lai (ST29), feng long (ST40), hua rou men (ST24), shui dao (ST28)
Bladder meridian of foot-Taiyang	Shen shu (BL23), pi shu (BL20), gan shu (BL18), ge shu (BL17), ci liao (BL32)
Extra points	Zi gong (EX-CA1), luan chao (TF2)
Kidney meridian of foot-Shaoyin	Tai xi (KI3), da he (KI12), heng gu (KI11), fu liu (KI7)
Liver meridian of foot-Jueyin	Tai chong (LR3), xing jian (LR2)
Du meridian	Bai hui (GV20), ming men (GV4), da zhui (GV14)
Large intestine meridian of hand-Taiyang	he gu (LI4)
Pericardium meridian of hand-Jueyin	Nei guan (PC6)
Gallbladder meridian of foot-Shaoyang	Dai mai (GB 26), xia xi (GB43)

**Table 2:** Ingredients of formula.

Formula	Composition
Cangfu Daotan Decoction (CDD)	White atractylodes rhizome (12 g), cyperus (12 g), pinellia rhizome (6 g), citrus (9 g), medicated leaven (6 g), bile arisaema (6 g), poria (9 g), bitter orange (12 g), fresh ginger (6 g), licorice root (6 g)
Xiao Yao San (XYS)	Licorice root (15 g), Chinese angelica (30 g), poria (30 g), tree peony bark (30 g), white atractylodes rhizome (30 g), bupleurum (30 g)
Jiawei Xiao Yao San (JWXYS)	Chinese angelica (3 g), white peony root (3 g), poria (3 g), white atractylodes rhizome (3 g), bupleurum (3 g), tree peony bark (1.5 g), gardenia (1.5 g), licorice root (1.5 g)
Danggui Shaoyao San (DSS)	Chinese angelica (10 g), white peony root (30 g), Sichuan lovage root (15 g), white atractylodes rhizome (15 g), poria (15 g), water plantain rhizome (15 g)
